# Insulin resistance and adipokines serum levels in a caucasian cohort of hiv-positive patients undergoing antiretroviral therapy: a cross sectional study

**DOI:** 10.1186/1472-6823-13-4

**Published:** 2013-01-26

**Authors:** Victoria Arama, Catalin Tiliscan, Adrian Streinu-Cercel, Daniela Ion, Raluca Mihailescu, Daniela Munteanu, Adriana Hristea, Stefan Sorin Arama

**Affiliations:** 1 Carol Davila University of Medicine and Pharmacy, Bucharest, Romania; 2 Prof. Dr. Matei Bals National Institute of Infectious Diseases, Bucharest, Romania

**Keywords:** Adipokines, Adiponectin, Leptin, Antiretroviral therapy, Insulin resistance, HIV

## Abstract

**Background:**

Insulin resistance is frequent in human immunodeficiency virus (HIV) infection and may be related to antiretroviral therapy. Cytokines secreted by adipose tissue (adipokines) are linked to insulin sensitivity. The present study is aimed to assess the prevalence of insulin resistance (IR) and its association with several adipokines, in a non-diabetic Romanian cohort of men and women with HIV-1 infection, undergoing combination antiretroviral therapy (cART).

**Methods:**

A cross-sectional study was conducted in an unselected sample of 89 HIV-1-positive, non-diabetic patients undergoing stable cART for at least 6 months. Metabolic parameters were measured, including fasting plasma insulin, and circulating adiponectin, leptin, resistin, tumor necrosis factor alpha (TNF-alpha) and interleukin-6 (IL-6) levels. Insulin resistance was estimated by measuring the Quantitative Insulin Sensitivity Check Index (QUICKI), using a cut-off value of 0.33. A linear regression model was fitted to QUICKI to test the association of IR and adipokines levels.

**Results:**

A total of 89 patients (aged 18–65, median: 28 years) including 51 men (57.3%) and 38 women (42.7%) were included in the study. Fifty nine patients (66.3%) were diagnosed with IR based on QUICKI values lower than the cut-off point. IR prevalence was 72.5% in men and 57.6% in women. The presence of the IR was not influenced by either the time of the HIV diagnosis or by the duration of cART. Decreased adiponectin and increased serum triglycerides were associated with increased IR in men (R=0.43, p=0.007). Hyperleptinemia in women was demonstrated to be associated with the presence of IR (R=0.33, p=0.03).

**Conclusions:**

Given the significant prevalence of the IR in our young non-diabetic cohort with HIV infection undergoing antiretroviral therapy reported in our study and the consecutive risk of diabetes and cardiovascular events, we suggest that the IR management should be a central component of HIV-infection therapeutic strategy. As adipokines play major roles in regulating glucose homeostasis with levels varying according to the sex, we suggest that further studies investigating adipokines should base their analyses on gender differences.

## Background

The introduction of combination antiretroviral therapy (cART) has significantly changed the course of human immunodeficiency virus (HIV) infection, increasing life expectancy but also leading to complex clinical and metabolic changes [1]. The term *lipodystrophy* was initially used to summarize these clinical and metabolic modifications [2]. The most important metabolic abnormalities observed in HIV-patients during cART include: insulin resistance (IR) with hyperinsulinemia and glucose intolerance [3], hypercholesterolemia with low levels of HDL-cholesterol, hypertriglyceridemia, lactic acidemia and hypercoagulopathy [4].

Lipodystrophy also associates fat redistribution: subcutaneous lipid stores wasting (Bichat’s fat pad, buttocks and extremities), central adiposity and fat accumulation in the dorsocervical region (lipohypertrophy) [2]. As patients with these metabolic abnormalities have relatively high rates of experiencing a cardiovascular event, there is growing concern about the potential damaging impact of cART in HIV-positive patients. Metabolic abnormalities may result from various and combined effects of cART antiretroviral drugs. Several studies have reported that almost all protease inhibitors (PIs) can cause IR, effects likely caused by the inhibition of glucose transport mediated by glucose transporter type 4 (GLUT-4) [5,6]. Antiretroviral drugs may also impair endocrine functions of adipocytes [7]. Adipose tissue synthesizes various adipokines and hormones, such as adiponectin, leptin, resistin, tumour necrosis factor-alpha (TNF-α), interleukins and release free-fatty acids [8]. Adiponectin and leptin may be involved in fat distribution and insulin sensitivity in HIV-infected patients [9]. Nucleoside reverse transcriptase inhibitors (NRTIs) may impair the secretion of adiponectin and thus may cause IR [10].

The aim of the study was to evaluate the prevalence of the IR and the association of the IR with clinical and biological variables, including serum adipokines, in a Caucasian HIV-infected non-diabetic population of men and women, undergoing cART.

## Methods

A cross-sectional research was performed in a Caucasian cohort of HIV-1-positive patients attending Prof. Dr. Matei Bals National Institute of Infectious Diseases from Bucharest, between June 2009 and June 2010. The protocol was approved by the Prof. Dr. Matei Bals National Institute of Infectious Diseases ethics committee (protocol no. 190/15.01.2009). An informed consent was obtained from each of the research subjects. The study was part of a national research grant (SLD-ART no. 62077/2008) on HIV infected patients. All consecutive non-diabetic patients who signed a written informed consent to participate in the study were enrolled. We included HIV-infected men and women, aged 18 years and over, undergoing stable cART for at least 6 months. The patients completed during the medical visit a standard questionnaire for demographic parameters and for personal and family medical histories. Body mass index (BMI) was defined according to WHO criteria [11]. Overweight was defined as a BMI ≥ 25 kg/m^2^ and obesity as BMI ≥ 30 kg/m^2^.

Blood samples were tested for: Adiponectin (BioSource ELISA, KAPME09), Resistin (BioSource ELISA, KAPME 50), Leptin (BioSource EASIA KAP2281), Tumor necrosis factor alpha - TNF-alpha (BioSource EASIA KAP 1751), Interleukin-6 (IL-6) (BioSource EASIA KAP 1261), CD4 T cells count (BD FACSCanto II flow cytometer), HIV viral load (Roche LightCycler® 480 Real-Time PCR System), fasting triglycerides, total cholesterol, low-density lipoprotein (LDL) cholesterol, high-density lipoprotein (HDL) cholesterol and fasting plasma glucose (dry slide technology, Vitros 950*,* Johnson & Johnson), fasting insulin (ELx50 Bioelisa Washer BioTek).

IR was determined using the Quantitative Insulin Sensitivity Check Index (QUICKI), calculated as *1/(log(fasting insulin μU/mL) + log(fasting glucose mg/dL)*[12].

In normal populations, mean QUICKI levels range from 0.37 to 0.39 [13]. Consistent with previous studies, QUICKI values were dichotomized at the cut-off of 0.33, lower levels signifying IR [13-15].

We also assessed the homeostatic model assessment – insulin resistance (HOMA-IR) index, first described by Matthews et al. in 1985 [16]. The formula used to calculate HOMA-IR was:

HOMA-IR = fasting insulin (μU/mL) × fasting plasma glucose (mmol/L)/22.

We assumed a HOMA-IR value of > 3 to define IR, consistent with previous studies investigating insulin resistance in Caucasian HIV populations [17].

### Statistical analyses

In order to test whether the samples came from normally distributed populations we used the Shapiro–Wilk test. Variables with gaussian distribution were expressed as means (+/−standard deviation [SD]). For non-normally distributed continuous data results were presented as median (interquartile range). Differences in means between groups were tested using independent-sample t-tests. We calculated the 95% confidence interval for the difference between the population means. Mann–Whitney test for non parametric continuous variables, Chi-square or Fisher’s exact test for categorical variables were used to test for statistical significance. Linear regression models were fitted to QUICKI score to test the association of IR with normally distributed continuous variables, included in the multivariate models either because of their known or suspected association with IR or based on an observed univariate association with QUICKI. Serum triglycerides, adiponectin, leptin, resistin, IL-6, TNF-alpha values were natural log transformed for the linear regression analysis. The correlation coefficient (R) was calculated to assess the linear correlation between the observed and model-predicted values of the dependent variables. We computed two-tailed p-values. Statistical significance was set at 5%, and all analyses were performed with SPSS® Statistics v.17.0, IBM®, New York, USA.

## Results and discussion

A total of 89 patients (age 18–65, median: 28 years) including 51 men (57.3%, age range: 18–63 years, median: 32 years) and 38 women (42.7% age range: 19–65 years, median: 21 years) were included in the study. The mean BMI was 23.6 (± 3.9) kg/m^2^. The median time from HIV diagnosis was 80.5 months and the median time on antiretroviral therapy was 72.5 months. Sixty five patients (73%) had a current PI-based cART regimen. Median serum levels of adiponectin, leptin, resistin, TNF alpha and IL-6 were 10.2 μg/mL, 1.4 ng/mL, 5.5 ng/mL, 10.3 pg/mL and 26.5 pg/mL, respectively.

Fifty nine patients (66.3%) were diagnosed with IR based on QUICKI values lower than the cut-off point. The overall prevalence of IR was 62.9%, based on HOMA-IR dichotomization. The median value of HOMA-IR was 3.77 (3.62). The patients were divided into Group 1 (patients without IR) and Group 2 (patients with IR). Group 1 patients had a mean QUICKI value of 0.35 (±0.02) and a median HOMA-IR of 1.84 (0.67) vs. 0.29 (±0.02) and 4.62 (3.48) in Group 2, respectively. The subject characteristics stratified by IR status are shown in Table 1. Significant differences were noted in total cholesterol, triglycerides and adiponectin serum levels between the two groups. The prevalence of IR was not significantly different between men and women. No significant differences were found between Group 1 and Group 2 in age, BMI, total time on cART or current protease inhibitor treatment.

**Table 1:** Clinical and biological characteristics of study patients by IR

**Characteristics**	**Total patients**	**Patients without IR (Group 1)**	**Patients with IR (Group 2)**	***P***
***N***	**89**	**30 (33.7)**	**59 (66.3)**	
**Age (years)**^**a**^	28 (22)	20.5 (13)	33 (25)	0.12
**Males**	51 (57.3)	14 (46.7)	37 (62.7)	0.14
**BMI (kg/m**^**2**^**)**	23.6 ± 3.9	22.6 ± 3.8	24.2 ± 3.8	0.3
**Fasting glucose (mg/dl)**	85.7 ± 13.7	82.3 ± 8.6	87.2 ± 15	0.12
**Haemoglobin A1c (%)**	4.85 ± 0.39	4.82 ± 0.25	4.86 ± 0.43	0.66
**Hypertension**^**b**^	23 (25.8)	8 (26.7)	15 (25.4)	1.00
**CD4 Tcell (cells/mm**^**3**^**)**^**a**^	440 (402)	525 (470)	428 (370)	0.38
**Patients with undetectable HIV viral load**	65 (73)	20 (66.7)	45 (76.3)	0.33
**Time on antiretroviral therapy (months)**^**a**^	72.5 (95)	80 (120)	70.5 (95)	0.21
**Time from HIV diagnosis (months)**^**a**^	80.5 (83)	92 (96)	76 (64)	0.36
**Current Protease Inhibitor treatment**	65 (73)	23 (76.7)	42 (71.2)	0.58
**Total cholesterol (mg/dl)**	192 ± 38.4	181.9 ± 33	199.6 ± 39.9	0.04*
**HDL cholesterol (mg/dl)**	43.9 ± 12.4	42.9 ± 10.5	44.3 ± 13.3	0.53
**LDL cholesterol (mg/dl)**	111.87 ± 35.4	107.3 ± 32.7	114.1± 36.8	0.39
**Triglycerides (mg/dl)**^**a**^	166 (129)	151.5 (79)	181 (169)	0.04*
**Dyslipidemia**^**c**^	71 (79.8)	24 (80)	47 (79.7)	1.00
**Hypertriglyceridemia**^**d**^	54 (60.7)	16 (53.3)	38 (64.4)	0.36
**Adiponectin (μg/mL)**^**a**^	10.2 (12.5)	16 (28.8)	9.4 (10.7)	0.00*
**Leptin (ng/mL)**^**a**^	1.4 (3.6)	1.8 (2.9)	1.3 (4.1)	0.53
**Resistin (ng/mL)**^**a**^	5.5 (3.6)	5.2 (5.1)	5.7 (3.4)	0.63
**TNF alfa (pg/mL)**^**a**^	10.3 (15.1)	11.9 (16.2)	9.7 (15)	0.62
**IL-6 (pg/mL)**^**a**^	26.5 (10.3)	26.6 (23.1)	26.5 (8.4)	0.52

Data are shown as mean ± SD or number (percent).

^a^ Results presented as median (interquartile range).

^b^ Hypertension defined as blood pressure ≥130/≥85 mm Hg or antihypertensive medication.

^c^ Dyslipidemia defined as elevation of one of the following: total cholesterol, LDL cholesterol, triglyceride serum concentrations or a decrease of HDL cholesterol.

^d^ Hypertriglyceridemia defined as serum triglycerides ≥150 mg/dl.

* p<0.05 Group 1 vs. Group 2.

p values based on *t*-test for equality of means or Mann–Whitney test for continuous variables, Chi-square or Fisher’s exact test for categorical variables.

In order to determine the relationship between laboratory parameters and IR, first we assessed the relationship of all individual variables with QUICKI score by fitting a linear regression model. The variables with univariate association to QUICKI were further tested to improve the model predictivity. After all variables were tested in the univariate analysis for the entire population, including BMI and age, only log transformed triglycerides serum levels (LOGTriglycerides) were significantly associated with QUICKI (R=0.24, p=0.01).

Fifity one men were enrolled in the study and 37 (72.5%) were diagnosed with IR. Male subject characteristics stratified by IR status are shown in Table 2. Significant between-group differences persisted in triglycerides and adiponectin serum levels. The significant variables selected to build the additive linear regression model in men, the estimate terms for the model and corresponding test statistics are provided in Table 3. Of the variables tested only log transformed adiponectin (LOGAdiponectin) and LOGTriglycerides had a significant effect on QUICKI values in our cohort of male subjects. Taken together, the analysis indicates that LOGAdiponectin with LOGTriglycerides were associated with QUICKI index (R=0.43, p=0.007) in males. The additive models that included age and BMI together with LOGAdiponectin and LOGTriglycerides lacked statistical significance.

**Table 2:** Biological characteristics of male and female study patients by IR

**Characteristics**	**Males**			**Females**		
	**Patients without IR (Group 1)**	**Patients with IR (Group 2)**	***P***	**Patients without IR (Group 1)**	**Patients with IR (Group 2)**	***P***
***N***	**14 (27.5)**	**37 (72.5)**		**16 (42.1)**	**22 (57.6)**	
**Adiponectin (μg/mL)**^**a**^	14.1 (31.8)	8.3 (10.5)	0.03*	18.1 (28.1)	14.4 (12.7)	0.18
**Leptin (ng/mL)**^**a**^	0.6 (1.2)	0.9 (1.2)	0.25	2.8 (2.3)	5.3 (8.8)	0.04*
**Resistin (ng/mL)**^**a**^	4.9 (3.3)	5.5 (3)	0.8	5.3 (6)	6.2 (4.6)	0.5
**TNF alpha (pg/mL)**^**a**^	13.8 (17.6)	10.4 (14.6)	0.65	10.4 (14.3)	8.9 (14.8)	0.62
**IL-6 (pg/mL)**^**a**^	35.8 (22.6)	27.7 (18.5)	0.46	23.9 (14.4)	25.2 (4.9)	0.22
**Total cholesterol (mg/dl)**	179.5± 33	197.6± 35.8	0.1	184± 34	202.9± 46.6	0.17
**LDL cholesterol (mg/dl)**	107.7± 34	115± 37.2	0.52	107± 32.6	112± 36.9	0.62
**HDL cholesterol (mg/dl)**	42.2± 12.5	40 ± 9.1	0.19	41.7± 8.5	48 ± 16	0.02*
**Triglycerides (mg/dl)**^**a**^	117.5 (84)	217 (157)	0.01*	167.5 (41)	165.5 (140)	0.96

Data are shown as mean ± SD or number (percent).

^a^ Results presented as median (interquartile range).

* p<0.05 Group 1 vs. Group 2.

p values based on *t*-test for equality of means or Mann–Whitney test for continuous variables, Chi-square or Fisher’s exact test for categorical variables.

**Table 3:** Multivariate analysis: effect of selected variables (LOGAdiponectin, LOGTriglycerides) on QUICKI (dependent variable) in male subjects

**Independent Variables**	**Unstandardized Coefficients (B)**	**Standardized Coefficients (beta)**	**95% Confidence interval for B**	**P**
**LOGAdiponectin**^**b**^	**0.009**	**0.278**	**(0.000; 0.001)**	**0.04**
**LOGTriglycerides**^**b**^	**−0.013**	**−0.261**	**(−0.026; 0.01)**	**0.03**

^b^LOGAdiponectin - log transformed adiponectin serum levels computed variable for linear regression.

^b^LOGTriglycerides - log transformed triglycerides serum levels computed variable for linear regression.

Of 38 women enrolled 22 (57.6%) were diagnosed with IR. Female laboratory characteristics stratified by IR status are shown in Table 2. Significant between-group differences were observed in HDL cholesterol and leptin serum levels. In women, of all variables tested in the univariate analysis, only log transformed leptin serum levels (LOGLeptin) were significantly associated with QUICKI (R=0.33, p=0.03) (Table 4). Other selected variables, including age and BMI, did not show any improvement of our additive model.

**Table 4:** Univariate analysis: effect of selected variables (LOGLeptin) on QUICKI (dependent variable) in female subjects

**Independent Variables**	**Unstandardized Coefficients (B)**	**Standardized Coefficients (beta)**	**95% Confidence interval for B**	**P**
**LOGLeptin**^**c**^	**−0.012**	**−0.339**	**(−0.023; 0.000)**	**0.03**

^c^LOGLeptin - log transformed leptin serum levels computed variable for linear regression.

IR appears when maintaining normal physiological values of glycaemia require insulin levels that exceed normal concentrations [18]. IR is associated with the development of diabetes and obesity [19]. Up to 20% of non-HIV western population may have IR [20,21]. Several studies reported a two-fold increase of cardiovascular risk compared to non-insulin resistant controls [22,23]. In the pre-cART era several cross-sectional studies reported slightly increased [24] or normal [25] insulin sensitivity when compared to uninfected controls. cART introduction led to increases in fasting insulin and decreases in insulin sensitivity, effects dependent both on the class of the antiretroviral used and the different antiretrovirals within each class [26,27]. Different studies have evaluated the prevalence of IR at 20 to 50%, in HIV-positive patients [28]. We noted higher levels of IR than reported by previous studies, independent of current treatment with PIs and of BMI, which may be attributed in part to our method of assessment. QUICKI has a very good correlation with the glucose clamp index of insulin sensitivity, which is considered the gold standard method for IR assessment [15]. IR presence in our population of HIV-positive young adults was not influenced either by the time from HIV diagnosis or to the duration of cART. This finding may suggest that the effect of cART on IR may not have a cumulative or long-term response, a similar result being reported also by other studies [29]. As IR presence is associated with an increased risk of diabetes, cardiovascular events, stroke and death, the reduction of its prevalence must be considered an essential therapeutic target in HIV-patients undergoing retroviral therapy.

Adiponectin is exclusively secreted from adipose tissue and has an important role as a mediator of peroxisome proliferator-activated receptor gamma action [30]. Adiponectin may increase insulin responsiveness of the glucose transport system in adipocytes through increased GLUT4 gene expression and increased GLUT4 recruitment to the plasma membrane [31]. Several studies have reported that adipocyte differentiation was perturbed both in vitro and in vivo as a direct effect of cART and the consecutive decrease in serum adiponectin levels was associated with insulin resistance [32,33]. We found an inverse correlation between adiponectin and IR in men, but not in females which partially concurs with previous studies. Vigouroux et al. (2003) have also reported that adiponectin was positively correlated with insulin sensitivity in a cohort of 131 HIV-infected men with PI-based cART regimens [9]. In a study on 237 Afro-Caribbean HIV-1-infected patients, published in 2011, Deloumeaux et al. found also an inverse correlation between adiponectin and IR in both men and women. After adjusting for clinical and metabolic parameters, the correlation between adiponectin and insulin resistance was observed only in women [34], a conclusion that contradicts our results and the previously mentioned study. Alterations of serum adiponectin concentration in relation to insulin resistance was also observed in a cohort of HIV-infected youths (aged 5.0 - 19.4 years), in a study published by Viganò et al. in 2011, which did not take into account sex differences but evaluated lipodystrophy. Low adiponectin concentration was associated to central fat and mixed lipohypertrophy and to signs of IR [35]. Other studies have also shown that adiponectin was inversely associated with IR, type 2 diabetes and obesity in HIV and non HIV-patients in without adjusting for gender differences [36,37].

Leptin is a 167-amino-acid adipokine secreted mainly by white adipose tissue. The levels of leptin are positively correlated with the amount of body fat [37]. Leptin regulates energy homeostasis and various neuroendocrine functions [38]. Our data showed higher levels of leptin in women with IR vs women without IR and a weak correlation between IR presence and leptin, observed in the linear regression model, with QUICKI as dependent and LOGLeptin as independent variable. Leptin did not contribute to an improvement of our linear model of IR in men. Other studies investigating this association have reported inconclusive results but very few have based their analyses on gender differences. Azzoni et al. in 2010 observed that serum leptin levels are inversely associated with HIV replication, independent of disease progression. In the same study, the authors reported a weak association between leptin levels and the Homeostatic Model Assessment for Insulin Resistance (HOMA-IR) [39]. Mynarcik et al. (2002) found that levels of leptin did not correlate with insulin sensitivity, in HIV-infected subjects with body fat redistribution [37]. In other study investigating adipokines and antiretroviral therapy related lipodystrophy, Tao et al. (2009) observed that leptin was positively correlated with the levels of total body fat, limb fat, and trunk fat, both in lipodystrophic and patients without body fat redistribution, but did not correlate with IR, and might serve only as a biomarker for total body fat mass [40]. Nagy et al. (2003) found a significant association between leptin concentrations and IR in patients with different morphological phenotypes. The lowest mean leptin levels were observed in patients with lipoatrophic changes. Hypoleptinemia was independently associated with IR in patients with lipoatrophy. Highest leptin concentrations were observed in patients with lipohypertrophy. Leptin levels in between the two extremes were observed in subjects with no body shape changes [41]. The role of leptin alterations in IR pathogenesis remains to be clarified by future long-term and prospective studies.

Despite the relative low number of patients, our study has several strengths. This is the first study in Romania to investigate adipokine dysregulation in association with HIV metabolic abnormalities during cART. The young age of our cohort is a distinct feature for the HIV epidemiology in Romania, compared with western European countries. The patients were probably infected parenterally in their childhood and adolescence between 1980–1990. QUICKI was used in order to diagnose IR, which is one of the most accurate surrogate indexes for determining insulin sensitivity in humans. Very few studies have evaluated untill now IR risk factors based on sex differences. Factors associated with IR, which have been lacking in previous studies, including the time from HIV diagnosis and the time on cART were measured and evaluated in our analyses.

Our study has some limitations. Correlations were small to moderate in magnitude. The association between IR and adiponectin/triglycerides in men and IR and leptin in women disappeared after including age and BMI in the regression models. This may be caused by BMI variations and changes in adiponectin with age that may contribute to IR pathogenesis and also by the low number of enrolled patients, with loss of statistical power as more variables were added. QUICKI was used to diagnose IR, a surrogate method, which has some limitations, such as the absence of a definite threshold for IR in Caucasian populations. QUICKI requires the quantification of fasting plasma insulin. Similar observations in clinical practice may be predicted with the use of International Diabetes Federation (IDF) or Adult Treatment Panel III (ATPIII) definitions of metabolic syndrome, which may provide cheaper ways for detecting patients at risk of diabetes and cardiovascular events. As leptin may be positively correlated with the levels of total body fat, limb fat, and trunk fat [41], the predictive value of our model might have been improved if we had included body fat redistribution in our analysis.

## Conclusions

We report a significant prevalence of IR in a young non-diabetic Caucasian population with HIV infection undergoing antiretroviral therapy that was independent of the time of the HIV diagnosis. Hypoadiponectinemia in men and hyperleptinemia in women was demonstrated to be associated with IR presence which brings new data to support the role of these cytokines in IR pathogenesis. As adipokines play major roles in regulating glucose homeostasis with levels varying according to sex, we suggest that further studies investigating adipokines should base their analyses on gender differences. In addition, new studies are needed to investigate the mechanisms contributing to the significant differences observed between men and women.

SLD-ART Study Group: Sorin Petrea, Otilia Benea, Mariana Mărdărescu, Monica Luminos, Dan Oţelea, Ana Maria Tudor, Simona Paraschiv, Viorica Leoveanu (deceased), Mihaela Rădulescu, Mihai Lazăr, Sergiu Costoiu, Ioana Olaru, Cristina Popescu, Cristina Loredana Benea, Ruxandra Moroti, Violeta Molagic, Viorica Poghirc.

## Abbreviations

cART: combined antiretroviral therapy; BMI: Body mass index; GLUT-4: glucose transporter type 4; HDL: high-density lipoprotein; HIV: human immunodeficiency virus; IL-6: Interleukin-6; IR: insulin resistance; LDL: low-density lipoprotein; NRTIs: Nucleoside reverse transcriptase inhibitors; PIs: protease inhibitors; QUICKI: Quantitative Insulin Sensitivity Check Index; TNF-α: tumour necrosis factor-alpha.

## Competing interests

The authors declare that they have no competing interests.

## Authors’ contributions

VA and ASC concieved the design of the study, coordinated the research study group and drafted the manuscript. CT and SSA performed statystical analyses and drafted the manuscript. RM and DM participated in patient enrollment and follow-up and reviewed the literature. AH and DI reviewed the paper before submission. VA, CT, ASC, DI, RM and SSA contributed equally to this work. All authors read and approved the final manuscript.

## Pre-publication history

The pre-publication history for this paper can be accessed here:

http://www.biomedcentral.com/1472-6823/13/4/prepub
